# Supplementary material for: Consequences of the Early Phase of the COVID-19 Pandemic for Home-Healthcare Recipients in Norway: A Nursing Perspective
Source: Healthcare (Basel). 2023 Jan 25;11(3):346. doi: 10.3390/healthcare11030346 (PMC9914130; doi:10.3390/healthcare11030346)
Supplement: Supplementary file 1 [file healthcare-11-00346-s001.zip › healthcare-2144150-supplementary.pdf]

Figure S1: Questionnaire, measurement of the consequences of the COVID-19 pandemic for home- healthcare service recipients.

To what extent do you experience that service recipients have experienced the following changes because of the COVID-19 situation?
1. Deterioration of condition<br>2. Poorer prognosis<br>3. Adverse events<br>4. Reduced level of functioning<br>5. Increased isolation/loneliness<br>6. Delayed diagnostics<br>7. Delayed follow-up/treatment<br>8. Less follow-up from the services than before the corona situation<br>9. More contact with the services than before the corona situation<br>10. Fewer physical meetings with the services<br>11. Increased burden on relatives/next of kin<br>12. Increased health concerns among service users/patients<br>13. Many cancelled consultations/treatments/contacts from the services<br>14. Many cancelled consultations/treatments/contacts from the users/patients<br>15. Many cancelled consultations/treatments/contacts from relatives<br>16. Less care after discharge/treatment/stay/contact<br>17. Has lost important support services<br>18. Has lost respite care services<br>19. Do not show up for consultations due to fear of becoming infected<br>20. Stopped taking medications because they are immunosuppressive<br>21. Several acute situations have emerged<br>22. Problems with access to medical supplies for chronically illness<br>23. Problems with access to medication
Response alternatives: (1) To a very large extent, (2) To a large extent, (3) To some extent, (4) To a small extent, (5) To a very small extent, (6) Not applicable.

Table S1. Pairwise correlation coefficients between the consequences (C1–C23)[1]. *Significance level 0.001.

	C1	C2	C3	C4	C5	C6	C7	C8	C9	C10	C11	C12	C13	C14	C15	C16	C17	C18	C19	C20	C21	C22	C23
C1	1																						
C2	**0.7432***	1																					
C3	**0.6024***	**0.6242***	1																				
C4	**0.6622***	**0.6251***	**0.6344***	1																			
C5	0.3557*	0.2999*	0.3019*	0.3506*	1																		
C6	0.3777*	0.4382*	0.4384*	0.3720*	0.3466*	1																	
C7	0.3623*	0.4078*	0.4077*	0.4096*	0.4158*	**0.7187***	1																
C8	0.3537*	0.3607*	0.3781*	0.3856*	0.3980*	0.4548*	**0.5904***	1															
C9	0.2451*	0.2965*	0.2330*	0.2138*	0.1222*	0.2365*	0.2247*	0.0813*	1														
C10	0.2129*	0.2462*	0.2601*	0.2536*	0.2909*	0.3065*	0.3811*	**0.5378***	0.0640*	1													
C11	0.3216*	0.3244*	0.3342*	0.3356*	0.3690*	0.3598*	0.4186*	0.4886*	0.1723*	**0.5120***	1												
C12	0.2817*	0.2592*	0.2626*	0.2451*	0.4372*	0.3566*	0.3556*	0.3500*	0.1607*	0.3455*	0.4478*	1											
C13	0.2876*	0.3158*	0.2911*	0.2781*	0.3333*	0.3689*	0.4068*	0.4689*	0.1200*	0.4278*	0.4087*	0.3584*	1										
C14	0.2674*	0.3089*	0.2659*	0.2783*	0.2678*	0.3435*	0.3458*	0.4082*	0.1919*	0.3906*	0.4171*	0.3696*	**0.5969***	1									
C15	0.2440*	0.2959*	0.2360*	0.2396*	0.2273*	0.2902*	0.3153*	0.3200*	0.1882*	0.3074*	0.3635*	0.3132*	**0.5055***	**0.6821***	1								
C16	0.3503*	0.3803*	0.3677*	0.3335*	0.2830*	0.4296*	0.4970*	0.4800*	0.1759*	0.3922*	0.3982*	0.3173*	**0.5304***	0.4554*	0.4570*	1							
C17	0.3306*	0.2918*	0.2927*	0.3476*	0.4284*	0.3624*	0.4176*	0.4473*	0.0920*	0.3397*	0.4280*	0.4020*	0.4387*	0.3817*	0.3683*	0.4519*	1						
C18	0.2942*	0.2793*	0.3087*	0.3190*	0.3711*	0.3038*	0.3618*	0.3934*	0.0991*	0.3055*	0.4065*	0.3533*	0.3741*	0.3594*	0.3453*	0.4042*	**0.5632***	1					
C19	0.2400*	0.2980*	0.2551*	0.2228*	0.2698*	0.3742*	0.3848*	0.3522*	0.2369*	0.3286*	0.3824*	0.3725*	0.4301*	**0.5345***	0.5100*	0.4176*	0.4179*	0.4319*	1				
C20	0.2474*	0.3082*	0.2711*	0.2202*	0.052	0.2828*	0.2326*	0.2143*	0.2494*	0.2339*	0.2341*	0.1583*	0.2468*	0.2822*	0.3131*	0.3513*	0.1631*	0.1954*	0.3534*	1			
C21	0.4032*	0.4508*	0.4344*	0.3634*	0.2297*	0.4001*	0.4310*	0.3772*	0.2359*	0.2938*	0.3330*	0.2701*	0.3810*	0.3729*	0.3699*	**0.5032***	0.3447*	0.3374*	0.3900*	**0.5195***	1		
C22	0.3069*	0.3618*	0.3442*	0.2947*	0.2102*	0.3955*	0.3906*	0.3238*	0.2127*	0.2715*	0.3210*	0.2781*	0.3485*	0.3364*	0.3250*	0.4493*	0.3218*	0.3209*	0.3974*	0.4497*	**0.5736***	1	
C23	0.2418*	0.2667*	0.2745*	0.2543*	0.1921*	0.2949*	0.3163*	0.2515*	0.1402*	0.1725*	0.2104*	0.2080*	0.2400*	0.2384*	0.2251*	0.3402*	0.2626*	0.2416*	0.2812*	0.3513*	0.4219*	**0.6419***	1

[1] C1=Deterioration of condition, C2=Poorer prognosis, C3=Adverse events, C4=Reduced level of functioning, C5=Increased isolation/loneliness, C6=Delayed diagnostics, C7=Delayed follow-up/treatment, C8=Less follow-up from the services than before the corona situation, C9=More contact with the services than before the corona situation, C10=Fewer physical meetings with the services, C11=Increased burden on relatives/next of kin, C12=Increased health concerns among service users/patients, C13=Many cancelled consultations/treatments/contacts from the services, C14=Many cancelled consultations/treatments/contacts from the users/patients, C15=Many cancelled consultations/treatments/contacts from relatives, C16=Less care after discharge/treatment/stay/contact, C17=Has lost important support services, C18=Has lost respite care services, C19=Do not show up for consultations due to fear of becoming infected, C20=Stopped taking medications because they are immunosuppressive, C21=Several acute situations have emerged, C22=Problems with access to medical supplies for chronically illness, C23=Problems with access to medication.
